# Culture Modulates the Brain Response to Harmonic Violations: An EEG Study on Hierarchical Syntactic Structure in Music

**DOI:** 10.3389/fnhum.2017.00591

**Published:** 2017-12-06

**Authors:** Haleh Akrami, Sahar Moghimi

**Affiliations:** ^1^Electrical Engineering Department, Ferdowsi University of Mashhad, Mashhad, Iran; ^2^Rayan Center for Neuroscience and Behavior, Ferdowsi University of Mashhad, Mashhad, Iran

**Keywords:** syntax, music, EEG, culture, hierarchical structures

## Abstract

We investigated the role of culture in processing hierarchical syntactic structures in music. We examined whether violation of non-local dependencies manifest in event related potentials (ERP) for Western and Iranian excerpts by recording EEG while participants passively listened to sequences of modified/original excerpts. We also investigated oscillatory and synchronization properties of brain responses during processing of hierarchical structures. For the Western excerpt, subjective ratings of conclusiveness were marginally significant and the difference in the ERP components fell short of significance. However, ERP and behavioral results showed that while listening to culturally familiar music, subjects comprehended whether or not the hierarchical syntactic structure was fulfilled. Irregularities in the hierarchical structures of the Iranian excerpt elicited an early negativity in the central regions bilaterally, followed by two later negativities from 450–700 to 750–950 ms. The latter manifested throughout the scalp. Moreover, violations of hierarchical structure in the Iranian excerpt were associated with (i) an early decrease in the long range alpha phase synchronization, (ii) an early increase in the oscillatory activity in the beta band over the central areas, and (iii) a late decrease in the theta band phase synchrony between left anterior and right posterior regions. Results suggest that rhythmic structures and melodic fragments, representative of Iranian music, created a familiar context in which recognition of complex non-local syntactic structures was feasible for Iranian listeners. Processing of neural responses to the Iranian excerpt indicated neural mechanisms for processing of hierarchical syntactic structures in music at different levels of cortical integration.

## Introduction

The human's brain is able to recognize syntactic violations in language and music (Patel et al., 1998; Kunert et al., 2015). Previous studies showed that music processing have similar recruited regions to language. In particular, increase in activation of Broca's area and prefrontal cortex was reported during music and language syntax processing in event related potentials (ERP), event related field (ERF) and functional magnetic resonance imaging (fMRI) studies (Stromswold et al., 1996; Levitin and Menon, 2003; Tillmann et al., 2003; Santi and Grodzinsky, 2010; Koelsch, 2011; Jeon and Friederici, 2013). The obtained results indicated that similar neural mechanisms and shared subsystems may be involved for processing syntax violation (Lashley, 1951; Fitch and Martins, 2014). In both language and music, elements are locally and non-locally dependent. Hierarchical syntax structure, which is one of the basic features of language capacity (Hauser, 2002), has also been described in the theory of music (Jackendoff and Lerdahl, 2006; Jackendoff, 2009). Hierarchical processing in music has been mainly investigated in the theory of harmony; certain rules about the arrangement of chord functions within harmonic sequences which express non-local hierarchical relationships between musical events (Rohrmeier, 2011). In a recent study using pure tone sequences it was demonstrated that non-musicians can process the recursive structure underlying the generation of musical fractals, and discriminate between well-formed hierarchies and foil categories (Martins et al., 2017). The first neural evidence for the processing of musical hierarchical syntactic structures with non-local dependencies was presented by Koelsch et al. (2013). The difference in brain responses to regular and irregular hierarchical structures, investigated through processing of ERPs corresponding to the final chord, manifested as an early bilateral frontal negativity that was maximal at around 200 ms, which was followed by a late negativity between 500 and 850 ms. Moreover, in an fMRI study it was demonstrated that abstract levels of hierarchical musical structures are encoded as one ascends from auditory regions to neighboring auditory areas, and then to the frontal cortex (Farbood et al., 2015).

Neuroimaging studies have not revealed distinctively different brain loci for processing of culturally familiar vs. culturally unfamiliar music (Morrison et al., 2003). Although music is present in all human societies, there exist within cultural structures, melodic signatures, and expectations that may mediate music perception amongst societies. The rules which dictate how the pitches and to some extent rhythms are allowed to be arranged constitutes the grammar of a musical culture. Native listeners of different musical systems implicitly learn the grammar of their music style through repeated exposure to various musical sequences (Levitin and Tirovolas, 2009). Moreover, presence of specific attributes and signatures in music, can make the processing of musical structures of that culture more automatic and efficient through creating a “feeling of knowing” (Nan et al., 2008). For instance, it is plausible to assume that listeners who grew up in a Western culture are more likely to be familiar with the rules and structures of tonal music and to be able to recognize presence of violations in complex musical structures, presumably due to repeated exposure to Western tonal music in everyday life. It is yet unknown whether listeners who are less familiar with Western tonal music can also recognize complex syntactic irregularities of tonal music vs. music of their own culture.

Iranian classical music is based on a number of modal scales and tunes. The common repertoire consists of more than two hundred short melodic movements (*gusheh*), which are in turn classified into seven modal systems called *dastgā h* (Farhat, 1990). Presence of meticulously ornamented notes and melodic and rhythmic elements in musical phrases are among the key aspects of Iranian classical music. Another distinct feature of Iranian music is its homophonic texture, where all voices coincide rhythmically. Although rules and structures of Western tonal music does not necessarily apply to Iranian classical music, overtime Iranian artists and elites have adopted the concepts of Western classical music, including harmony, in their compositions (Fakhrodini, 2015). Hierarchical structures are evident in different musical cultures (Stevens, 2012). Non-local dependencies and hierarchical structures are also present in Iranian classical music. As an example readers are referred to the highly popular Iranian song *Morghe Sahar* which was composed in *Mahoor* (name of an Iranian musical *dastgāh*) by Morteza Neydavoud in 1927.

The aim of the present study is to determine whether there exists a “first music response” to complex hierarchical structures in music. Previously, engagement of a brain mechanism in perception of nested non-local dependencies in music has been demonstrated in Western listeners (Koelsch et al., 2013). Here, we seek to investigate whether the aforementioned mechanism is affected by cultural preferences and musical systems. To investigate the above mentioned issue, we replicated a previously reported experiment (Koelsch et al., 2013) to study the processing of hierarchical syntactic structures in music among Iranian musicians and non-musicians. However, in the current study we used one chorale by J. S. Bach (BWV 373) and one homophonic Iranian excerpt with identical hierarchical syntactic structure to BWV 373, composed for the purpose of the current study. We hypothesized that Iranian non-musicians (vs. musicians) could not recognize the violation of non-local prolonged dependencies present in BWV 373, and that the previously reported neural responses (in terms of both early and late ERP components) to irregular hierarchical structures in BWV 373 (Koelsch et al., 2013) would not be present in our non-musicians. However, the relatively familiar context of the Iranian excerpt would help them process the syntactic structure more efficiently through creating a “feeling of knowing” and therefore recognize trials which included violations of prolonged dependencies. To investigate the neural substrates we tested whether final chords of trials with hierarchical irregularities (for both BWV 373 and the Iranian excerpt, hence both culturally unfamiliar and familiar music) would evoke significant brain responses compared with the hierarchically regular versions. Toward this endeavor, we used electroencephalography (EEG) and performed ERP, time-frequency, and phase synchrony analysis over the final chords of hierarchically regular/irregular versions.

## Materials and methods

### Participants

Twelve musician (age range: 29 ± 6 years) and 12 non-musicians (age range: 24 ± 1 years) participated in this study. The numbers of women and men in each group were equal (six females in each grou). Admitted musicians had at least 10 years of training on Iranian music (on either string or keyboard instruments). Non-musicians have received none or less than 1 year of training on any music instrument. All participants were right-handed, had normal hearing (according to self-reports), and had no history of any neurological/psychological disorder. They reported normal nocturnal sleep patterns (7–9 h starting from 10 p.m. to 12 a.m.) for the week before the experiment. They had not used caffeine, nicotine, or energy drinks and had not performed excessive exercise in the 24 h before the experiment. Written informed consent was obtained from all subjects; the study was conducted according to the Declaration of Helsinki. The Ethics Committee at the Ferdowsi University of Mashhad approved the study protocol. All volunteers were rewarded with either monetary compensation or course credits.

### Stimulus

**Figure 1:**
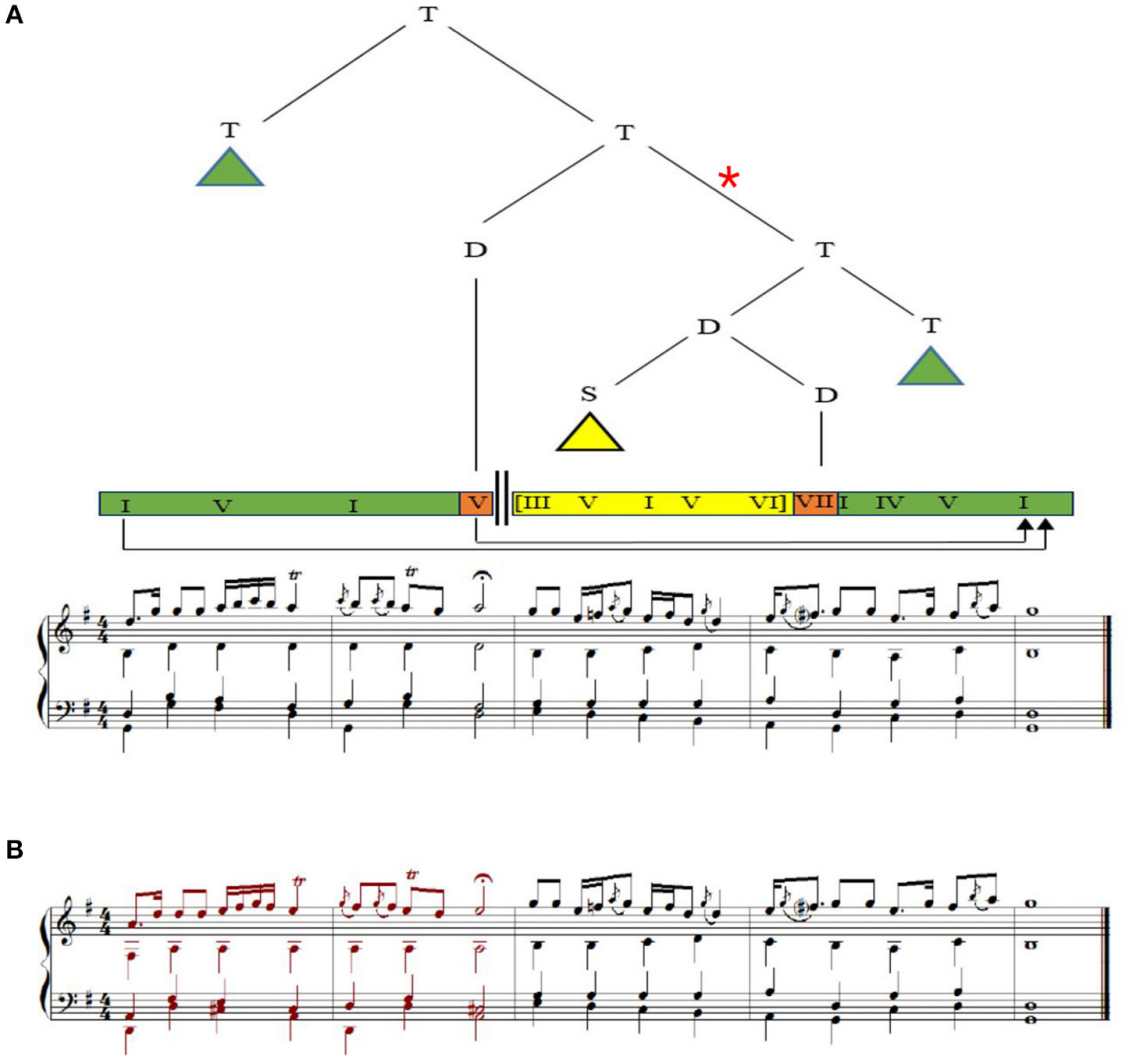
**(A)** Original version of the Iranian excerpt, and its tree structure of harmonic dependencies. T, tonic region; D, dominant region, S, subdominant region. Roman numerals represent scale degrees. Arrow indicates harmonic prolonged dependency between the initial and final tonic as well as between the half cadence and the final chord. Triangles indicate dependency structures not shown here. The two thick parallel vertical lines indicate that the local dominant has not immediately resolved in a tonic chord. Green, orange, and yellow rectangles represent scale degrees relative to the local key. **(B)** Modified version of the Iranian excerpt created by transposing the first phrase down a forth. Harmonic dependency between initial and final tonic is not fulfilled in this version. The connection marked by a red asterisk has been violated and does not exist in this version.

Our stimuli comprised of two excerpts; two phrases of a choral by J.S. Bach (BWV 373) (Koelsch et al., 2013) and an Iranian excerpt (as shown in Figure 1) with the identical hierarchical syntactic structure to BWV 373 according to the Generative Theory of Tonal Music (GTTM) (Cady, 1983) and the Generative Syntax Model (GSM) (Rohrmeier, 2011). The Iranian excerpt was created by a professional Iranian composer with the aim of eliciting a sense of familiarity (without reference to episodic memory) toward the music piece while keeping the hierarchical syntactic structure intact. During the composition process melodic signatures (e.g., presence of ornaments), rhythmic structures (e.g., uneven rhythms), and concepts of modal structures (including the concept of tetrachords, or *dângs*) of Iranian music were employed. Subjective assessment of Iranian listeners were considered during the composition process, although not reported in this study. In both pieces there were two phrases consisting of five bars, with the first phrase ending with a half cadence, and the second phrase beginning with a chord other than the tonic and ending on the original tonic by means of an authentic cadence. Hence, the final chord of each original version hierarchically prolonged the first chord of the excerpt, according to the GTTM and GSM. Modified versions of both Western and Iranian excerpts were created exactly the same as what was performed by Koelsch et al. (2013); by transposing the first phrase down a fourth (Figure 1B). By performing the aforementioned process, the final chord of the second phrase of each modified version no longer prolonged the first chord of the piece, and therefore did not close the dominant established by the first phrase. Since the second phrases of the original and modified versions were exactly the same, the local probabilities for the transition between penultimate and final chords were equal in both versions. Through the aforementioned manipulation we created irregularity in the hierarchical structure, while keeping the local structure intact. Later, the two original versions and the two modified versions were transposed to the 12 major keys, and exported as wav files with a piano sound and a tempo of 100 beats per minute (see Supplementary Material for audio files of original excerpts as well as samples of versions with hierarchical irregularities). Again similar to what was previously performed (Koelsch et al., 2013) a timbre detection task was created by producing a new modified version, different from what was explained above; one voice of one bar of 25 different stimuli (out of 48 total stimuli: 2 excerpts × 2 versions × 12 keys) was randomly played with an instrument (bassoon) other than piano. Participants were informed about the presence of these deviations and their task was to detect them. The trials with timbre variations were excluded during EEG analysis.

### Experimental procedure

**Figure 2:**
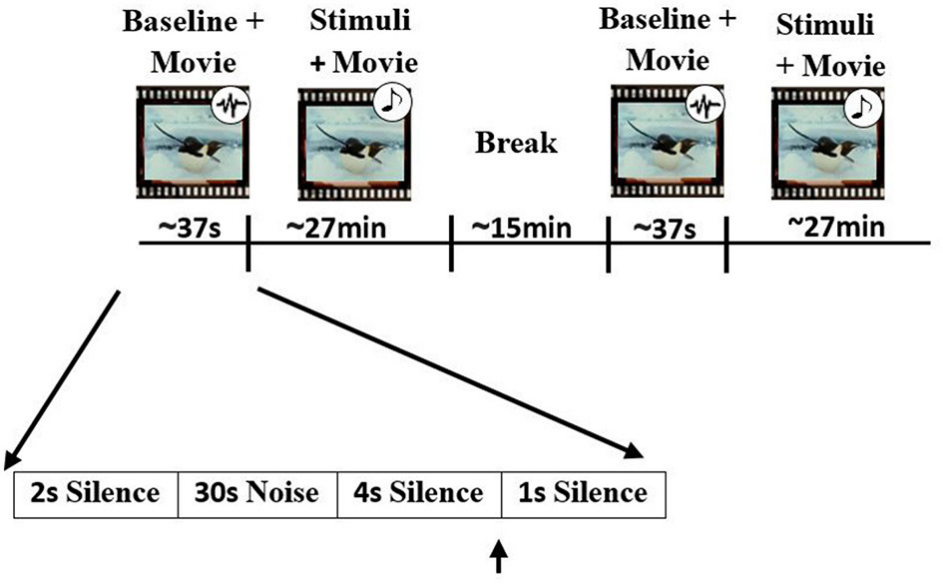
Experimental procedure. Participates were asked to listen to pieces and detect timbre deviants while watching the silent movie (March of Penguins, Warner, ASIN B000BI5KV0). 265 stimuli were presented randomly in two parts with a 15 min rest interval in between. Baseline recording was performed at the beginning of each part using 30 s of brown noise. Black arrows represent the times at which a short beep sound was played.

All participant started the experiment by filling out the musical experience and mood questionnaires. The EEG session consisted of two parts separated with a 15 min rest interval. Each part included a 30 s brown noise interval before excerpt presentation. Participants were instructed to listen to the stimuli (at 60 dB sound pressure level) through headphones while watching a silent movie (March of the Penguins, Warner, ASIN B000BI5KV0) and to press a button whenever they detected a timbre deviant. The 48 stimuli without timbre deviants (consisting of different versions of both Western and Iranian excerpts) were presented five times, randomly intermixed with 25 sequences containing a timbre deviant (265 stimuli in total). Each stimulus presentation was followed by 1 s silence (Figure 2). After the EEG session, 24 excerpts of the previously presented stimuli (six original and six modified versions for each of the two excerpts, with each stimulus in a different key) were presented to the participants. Right after exposure, subjects were asked to rate the excerpts on conclusiveness of the final chord, valence, and arousal of the excerpt and how much the presented piece sounded Iranian. Scales ranged from one (low conclusiveness, low valence, low arousal, and not an Iranian piece) to nine (high conclusiveness, high valence, high arousal, and totally an Iranian piece).

### EEG recordings and preprocessing

EEG Data were acquired using a g.USBamp (g.tec Medical Engineering GmbH, Austria) from 28 electrodes (AF3, AF4, F1, F2, F3, F4, F5, F6, FC1, FC2, FC3, FC4, C1, C2, C3, C4, CP1, CP2, CP3, CP4, CP5, CP6, P1, P2, P3, P4, P5, P6), according to the 10–20 system, with a sampling frequency of 512 Hz. Three electrodes were used for recording the electrooculogram (EOG); two electrodes were placed above and below the left eye to record the vertical EOG, and another electrode was positioned at the outer canthus of the left eye to record the horizontal EOG. A band pass 0.5–60 Hz filter was applied to remove low and high frequency artifacts from the EEG signals. EEG data were later re-referenced to the algebraic mean of the left and right mastoids. Artifacts (e.g., eye-blink eye-movement, and muscle activity) were then removed by employing Independent Component Analysis (ICA) using EEGLAB toolbox (Delorme and Makeig, 2004). Next, data were filtered with a 25-Hz low pass filter (FIR, 550 points) to remove the remaining high frequency noise. The preprocessed data were later epoched. Finally, epochs were excluded if within a moving window of 600 ms the standard deviation of amplitude exceeded 25 μV, or any sampling point exceeded ±75 μV at any electrode location. After exclusion of epochs with artifacts the average number of remaining epochs among subjects was 54.96 ± 3.68 and 55.58 ± 3.66 for the original and modified versions of the Western excerpt and 55.25 ± 4.37 and 55.38 ± 4.28 for the original and modified versions of the Iranian excerpt, respectively.

### ERP analysis

For each subject the remaining epochs were averaged for the last chord separately for each modified and original version of all excerpts from −200 to 1,200 ms relative to the onset of the final chord and demeaned using a baseline from −200 to 0 ms. For statistical analysis of ERPs mean amplitude values were computed for four regions of interest (ROIs); left anterior (LA, including: AF3, F1, F3, F5, FC1, FC3), right anterior (RA, including: AF4, F2, F4, F6, FC2, FC4), left posterior (LP, including: CP1, CP3, CP5, P1, P3, P5), and right posterior (RP, including: CP2, CP4, CP6, P2, P4, P6).

### Time-frequency analysis

Energy values of Time-Frequency Representations (TFR) of single trial data were obtained by computing the squared norm of the complex Morlet wavelet transform (Tallon-Baudry et al., 1997; Ruiz et al., 2009)

$$\begin{array}{l} E\left(t,f\right)=||w\left(t,f\right)|{|}^{2} \end{array}$$

where *w*(*t, f*) is the complex wavelet transform of each epoch (from −200 to 1,200 ms with respect to the onset of the final chord). For visualization, for each subject and electrode location a frequency dependent normalization was performed with respect to the average TFR value of brown noise over windows of 1,200 ms (with 200 ms overlap). For statistical analysis normalization was performed for each subject, each electrode location, and each frequency band [*f*, namely, theta (4–8 Hz), alpha (8–13 Hz), and beta (13–25 Hz)] with respect to the corresponding average TFR value of brown noise over the corresponding frequency band and windows of 1,200 ms (with 200 ms overlap).

$$\begin{array}{l} E_{norm}\left(t,f\right)=\frac{E\left(t,f\right)}{\mu_{base}\left(f\right)} \end{array}$$

where μ_*base*_, is the mean of the TFR energy corresponding to listening to brown noise.

### Phase synchrony analysis

First EEG data were filtered to obtain signals in three frequency bands: theta, alpha, and beta using FIR filters from EEGLAB toolbox. To measure phase synchrony, the method introduced by Tass et al. (1998) and Gokmen and Vlasov (2016) was employed. First, for *x*(*t*) the analytic signal was calculated as

$$\begin{array}{l} x_{an}\left(t\right)=x\left(t\right)+ix_{HT}\left(t\right) \end{array}$$

where *x*_*HT*_(*t*) is the Hilbert Transform of *x*(*t*). Next, instantaneous amplitude, *A*_*x*_(*t*), and phase, φ_*x*_(*t*), of *x*(*t*) were calculated

$$\begin{array}{l} A_{x}\left(t\right)=\sqrt{x^{2}\left(t\right)+x_{HT}^{2}\left(t\right)}\\ \varphi_{x}\left(t\right)=tan^{-1}\left(\frac{x_{HT}\left(t\right)}{x\left(t\right)}\right) \end{array}$$

The distribution function of phase difference between two signals *x*_i_(*t*) and *x*_j_(*t*), ϕ_*ij*_(*t*) = |φ_*i*_(*t*) − φ_*i*_(*t*)|, was estimated, and its deviation from the δ-distribution (corresponding to perfect synchrony) was computed using

$$\begin{array}{l} \rho =\frac{E_{max}-E}{E_{max}} \end{array}$$

where *E* is the Shannon's entropy of the distribution function ϕ_*ij*_(*t*) mod 2π. The maximum entropy *E*_*max*_ (corresponding to a uniform distribution, and hence no phase relation) is given by ln(M), *M* = *e*^(0.626+0.4*ln*(*L*−1)^, *L* being the number of time samples (Otnes and Enochson, 1972). ρ is confined between 0 and 1. The higher the value of ρ between two channels, the stronger the degree of phase synchronization. Employing the described method the phase synchrony index was calculated for each pair of electrodes.

For each trial, the final chord was divided into two windows of 600 ms and the value of ρ, corresponding to each possible electrode pair was computed for each of the aforementioned windows and then averaged over trials for each subject, version, and excerpt, separately. For normalization, both the first and second 30 s brown noise intervals were divided into epochs of 600 ms with 100 ms overlap and the value of ρ was calculated for each epoch and later averaged over epochs. Finally, the phase synchrony index was normalized with respect to ρ_*noise*_.

## Results

### Behavioral results

In average, musicians and non-musicians detected 99 and 98% of trials with timbre deviants, respectively, demonstrating that both groups performed the instructed task correctly. As expected the Iranian excerpts sounded more Iranian compared to the chorales [*F*_(1, 20)_ = 31.50, *p* < 0.01]. Moreover, conclusiveness ratings for the original excerpts were higher than those of the modified versions. Repeated measure ANOVAs performed for the conclusiveness ratings of both excerpts with factors version (original, modified) and group yielded a significant effect of version [Western: *F*_(1, 20)_ = 4.47, *p* < 0.05, Iranian: *F*_(1, 20)_ = 10.24, *p* < 0.01], with no interaction between factors. For the Western excerpt ANOVAs performed for valence and arousal ratings did not indicate any significant effect for the aforementioned factors. However, for the Iranian excerpt ANOVA performed for valence ratings revealed an effect of version [*F*_(1, 20)_ = 12.46, *p* < 0.01]; the original excerpts were rated more pleasant compared to the modified versions by both groups (Table 1).

**Table 1:** Behavioral results.

	**Western**	**Iranian**
Conclusiveness	**−0.26 (0.13)**	−0.36^*^ (0.11)
Valence	−0.20 (0.18)	−0.58^*^ (0.17)
Arousal	−0.27 (0.15)	−0.04 (0.23)
		

Mean values (with standard error in parentheses) of rating differences between versions (original subtracted from modified versions). Bold font indicates that rating differences between original and modified versions were statistically significant (p < 0.05).

* *p < 0.01*.

### ERP results

**Figure 3:**
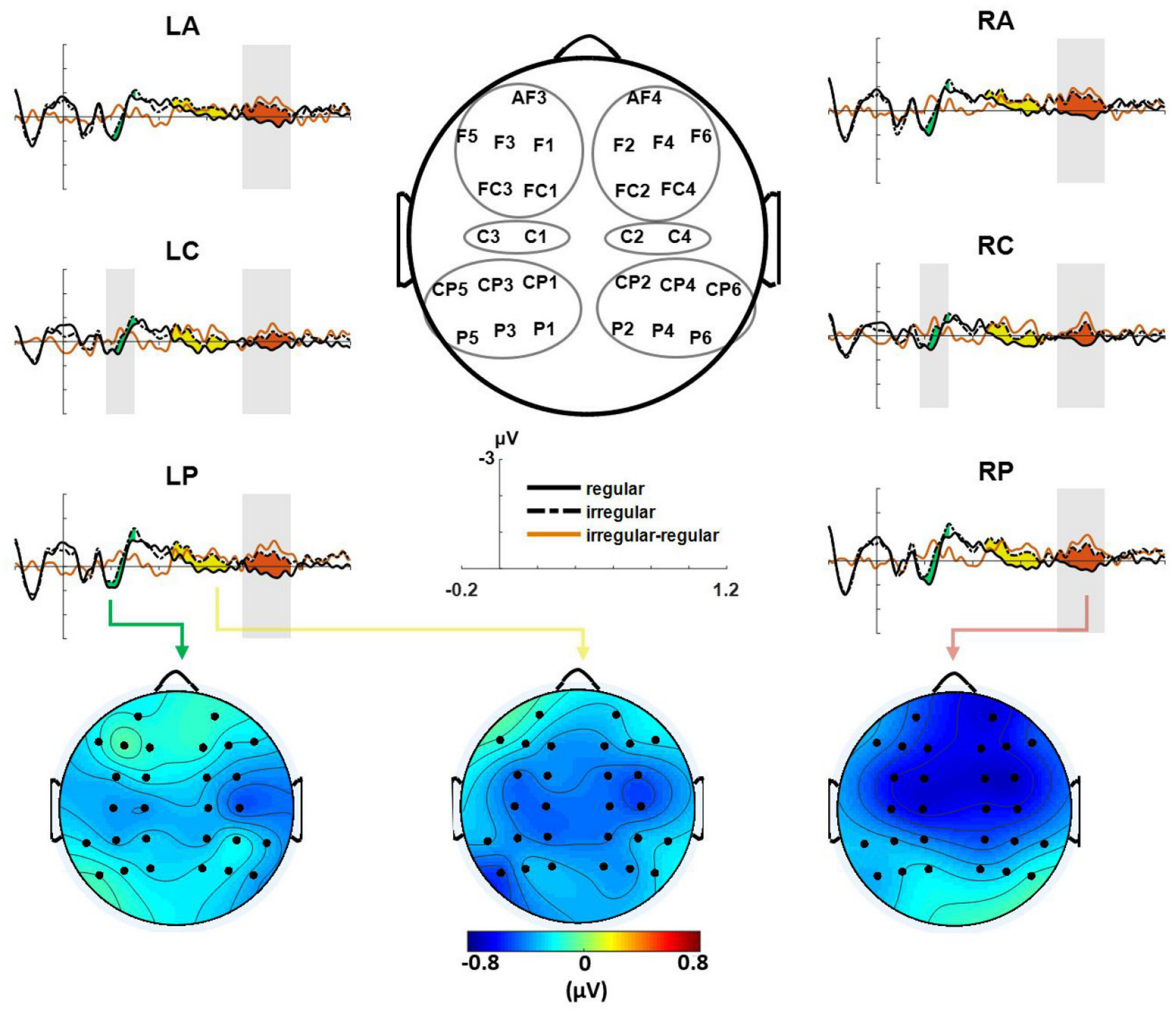
Event-related brain potentials (ERPs) evoked by the final chords in ROIs are shown, modified version (difference illustrated in red) elicited an early negativity (maximal at around 230 ms) and two late negativities from 450 to 700 ms and from 750 to 950 ms. Gray-shaded areas indicate time windows with a significant effect in statistical analysis. Bottom figures show the scalp distribution of the early **(Left)**, the first late **(Middle)** and the second late negativity **(Right)** evoked by modified versions (shown as original subtracted from modified versions).

For the Iranian excerpt, ERP analysis of the final chord for the modified versions (compared with the original versions) revealed an early negative brain electric response peaking around 230 ms at central areas over both hemispheres. This effect was followed by two later negativities from 450 to 700 ms over the same brain areas and also from 750 to 950 ms which manifested globally as shown in Figure 3. For statistical analysis of ERPs mean amplitude values were computed for four ROIs (see also Figure 3); left anterior (LA, including: AF3, F1, F3, F5, FC1, FC3), right anterior (RA, including: AF4, F2, F4, F6, FC2, FC4), left posterior (LP, including: CP1, CP3, CP5, P1, P3, P5), and right posterior (RP, including: CP2, CP4, CP6, P2, P4, P6). Next repeated measure ANOVAs were conducted for time windows 180–300, 450–700, and 750–950, with the within-subject factors version (original, modified), anterior–posterior (anterior, posterior), and, hemisphere (left, right), as well as the between-subjects factor group (musicians, non-musicians). The ANOVA for the time-window 750–950 ms, revealed an effect of version [*F*_(1, 22)_ = 5.68, *p* < 0.05], with no significant difference between groups or interaction between factors. A follow-up repeated measure ANOVA for central ROIs (left central, LC including: C1, C3, and right central, RC including: C2, C4) with the within-subject factors version, and hemisphere, as well as the between-subjects factor group yielded a significant effect of version in time windows 180–300 ms [*F*_(1, 22)_ = 4.45, *p* < 0.05] and 750–950 ms [*F*_(1, 22)_ = 8.163, *p* < 0.01] with no significant effect of group or interaction between factors. There was a marginally significant main effect of version for the time window 450–700 ms [*F*_(1, 22)_ = 3.24, *p* = 0.078] in the central ROIs (see also Supplementary Table 1).

The Results were similar for the Western excerpts, in terms of the presence of an early negativity at 180–300 ms and a late negativity at 450–700 ms, however the observed differences in the aforementioned time windows did not verify as significant. Moreover, no significant effect was observed for any of the factors (Supplementary Figure 1 and Supplementary Table 2). Hence, hereafter further analyses were performed only for different versions of the Iranian excerpt.

### Time-frequency results

**Figure 4:**
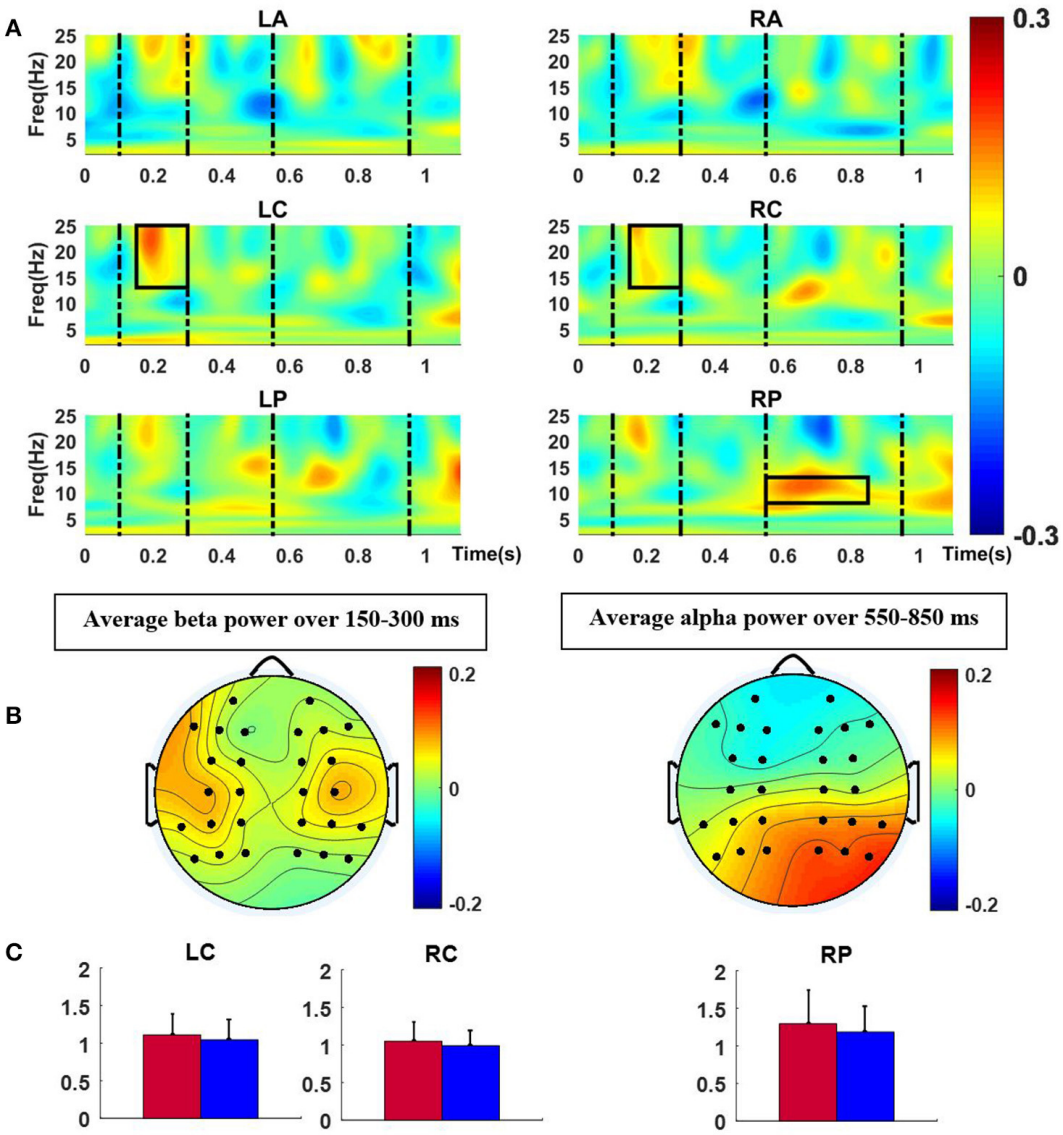
**(A)** Normalized TFR energy of modified-original versions for the final chord, dash lines represent time windows of interest (100–300, 550–950 ms) and Black rectangles indicate the time-frequency intervals with significant effects. **(B)** Scalp distributions of the significant differences between modified and original versions, averaged over time and frequency for the rectangles in **(A)**. **(C)** Bar plots of mean power (and standard deviation) in the TFR windows of significant effect (left: 13–25 Hz and 150–300 ms, and right: 8–13 Hz and 550–850 ms) for each version (blue and red colors represent original and modified versions, respectively).

For the final chord, the mean TFRs over six ROIs (same as those employed for ERP analysis) were computed as shown in Figure 4. For each ROI, repeated measure ANOVAs were conducted with factors version and group for the three frequency bands in a moving window of length 100 ms with 50% overlap inside the intervals 100–300, and 550–950 ms, which were selected after inspection of ERP results and observation of time windows with significant effects (180–300 and 750–950 ms). As Table 2 indicates, for the time window 150–300 ms, mean beta band TFR energy of the central regions was found to be higher for the modified versions compared to the original excerpts; the difference was statically significant [LC: *F*_(1, 21)_ = 5.50, *p* < 0.05, RC: *F*_(1, 21)_ = 5.45, *p* < 0.05], with no effect of group or interaction between factors. For the time window 550–850 ms, the modified versions elicited more TFR energy than the original versions in RP in the alpha frequency range. The difference reached significance for the factor version [*F*_(1, 21)_ = 6.81, *p* < 0.05].

**Table 2:** Significant effects of version for TFR energy of the final chord.

**Time window**	**Frequency band**	**Region**	***F*_(1, 21)_**	**Difference**
150–300 ms	Beta	LC	4.50	0.06 (0.03)
	Beta	RC	4.44	0.05 (0.01)
550–850 ms	Alpha	RP	6.81	0.11 (0.04)

*Difference is reported as modified-original (with standard error in parentheses)*.

### Phase synchrony results

**Figure 5:**
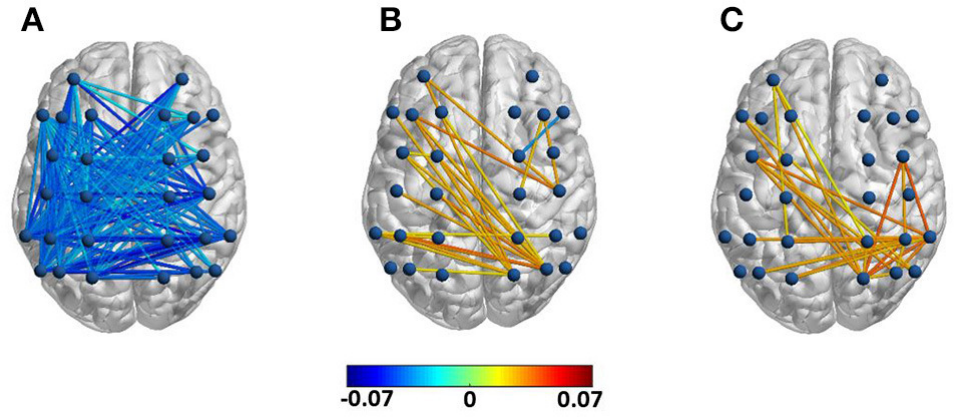
Difference in phase synchrony between modified and original versions (modified—original) for each electrode pair with significant effect of version. **(A)** Difference in alpha band phase synchrony in the first half for the factor version, **(B)** difference in theta band phase synchrony in the second half for the factor version, **(C)** difference in alpha band phase synchrony in the second half for the factor version.

As Figure 5 shows, created using BrainNet Viewer (Xia et al., 2013), for the first half of the final chord, anterior-posterior and intra-posterior phase synchrony (depicted in terms of inter-electrode index values) decreased for the modified versions compared to the original versions in the alpha frequency range. This was followed by an increase in both theta and alpha band phase synchrony between left anterior and right posterior electrodes as well as an increase in the local intra-posterior (only in the right hemisphere) alpha band phase synchrony in the second half of the final chord. The depicted inter-electrode connections were drawn after performing separate repeated measure ANOVAs for each frequency band of interest with factors version and group on each pair of electrodes. We did not observe robust significant phase synchrony differences in other frequency bands nor, notably, between other pairs of channels not depicted in Figure 5. Moreover, no significant effect of group was observed. For further statistical analysis electrodes were grouped into four ROIs, LA, RA, LP, and RP and inter-regional connectivity indices were computed as the average values of the inter-electrode indices belonging to each of the aforementioned ROIs. An ANOVA with the factors version and group revealed a significant effect of version in the alpha band phase synchrony between LA-LP [*F*_(1, 21)_ = 17.09, *p* < 0.01], LA-RP [*F*_(1, 21)_ = 5.73, *p* < 0.05], RA-LP [*F*_(1, 21)_ = 11.52, *p* < 0.01], and LP-RP [*F*_(1, 21)_ = 7.38, *p* < 0.05] for the first half of the final chord. In additions, for the second half of the final chord, ANOVAs conducted over the inter-regional phase synchrony indices reached significance for the factor version for LA-RP [*F*_(1, 21)_ = 5.68, *p* < 0.05] in the theta frequency range and for RP-RP [*F*_(1, 21)_ = 9.67, *p* < 0.01] in the alpha frequency range (see also Table 3).

**Table 3:** Significant effects of phase synchrony in the final chord.

**Time**	**Frequency band**	**Region**	***F*_(1, 21)_**	**Difference**
First half	↓Alpha	LA-LP	17.09	**−0.038 (0.009)**
	↓Alpha	LA-RP	5.73	−0.025 (0.010)
	↓Alpha	RA-LP	11.52	**−0.032 (0.009)**
	↓Alpha	LP-RP	7.38	−0.041 (0.016)
Second half	↑Theta	LA-RP	5.68	0.021 (0.009)
	↑Alpha	RP-RP	9.67	**0.022 (0.007)**

*Difference is reported as modified-original (with standard error in parentheses). Bold font indicates p < 0.01*.

## Discussion

Both ERP and behavioral results demonstrated that while listening to culturally familiar music, subjects could comprehend whether or not the final chord prolonged the first chord, or in other words whether or not the hierarchical syntactic structure was fulfilled. For the Western excerpt however, subjective ratings of conclusiveness were only marginally significant (*p*-value close to 0.05) and the difference in the ERP component fell short of significance. The motivation for our choice to adopt this previously introduced study design (Koelsch et al., 2013) and for investigating the role of culture in processing of non-local dependencies in music rather than local dependencies or other relatively simpler syntactic structures was the assumption that the role of culture was more likely to manifest in processing of complex syntactic structures. Although musicians performed better in the behavioral study no group effect was observed in either of the behavioral and neurophysiological results. (i) Perhaps in the culturally familiar context of the Iranian excerpt multiple exposure (during the experiment) to the complex hierarchical structure led to the establishment of equal sensitivity to non-local dependencies for both groups. (ii) Our musicians could have been relatively more attuned to the structure of the Iranian excerpts due to their training on Iranian classical music.

The violation of prolonged dependencies in the Bach's chorale did not led to significant ERP responses. However, during ERP analysis of the brain electrical response corresponding to the Iranian excerpt, processing of irregularities in the hierarchical syntactic structure elicited both early and late ERP responses. Given the aforementioned information the question arises whether more pronounced neural and consequently behavioral responses to the violation of non-local dependencies for the Iranian excerpt was due to its culturally familiar theme which provided a feeling of ease or knowing for the native listeners or it was the relatively simpler harmonic and rhythmic structure of the Iranian piece, compared to the chorale, which caused the observed neural and behavioral responses. To address this issue we performed a follow up behavioral study. Twenty eight new Iranian non-musicians (14 females, age range: 25 ± 3 years) participated in this study. Considerations for subjects' inclusion criteria were the same as those previously described. Fourteen of the subjects performed the previously described behavioral task (section Experimental Procedure). The other 14 performed the exact same task with the exception that instead of the original Bach's chorale they listened to a structurally simpler homophonic version of the excerpt (Audio File A3, see also Supplementary Figure 2), modified for the purpose of the current study and with its hierarchical structure kept intact. Considerations for preparing the stimuli for the simplified version along with its variations were the same as those described in section Stimulus. We aimed to investigate whether naïve Iranian listeners can behaviorally identify the presence of irregularities in the simplified version of the Bach's chorale. The results are presented in Table 4. For the first group (who listened to the original version of Bach's chorale) ratings of conclusiveness and valence for the Iranian excerpt yielded a significant effect of version [*F*_(1, 12)_ = 4.95, *p* < 0.05, and *F*_(1, 12)_ = 8.02, *p* < 0.05, respectively]. For the Western piece however neither of the ratings reached significance. For the second group once again ratings of conclusiveness and valence for the Iranian excerpt yielded a significant effect of version [*F*_(1, 13)_ = 4.84, *p* < 0.05, and *F*_(1, 13)_ = 6.74, *p* < 0.05, respectively]. Although the mean difference of conclusiveness ratings between the original and modified versions of Bach's chorale was relatively larger for the second group, it did not reach significance. This experiment demonstrated that Iranian non-musicians could not recognize the violation of prolonged dependencies in the original as well as the simplified version of Bach's chorale, with a homophonic texture and relatively simpler structure. For both groups however the aforementioned deviation was recognized for the Iranian excerpt. It may be argued that the working memory load required to establish and maintain a representation of the hierarchical structure was the source of the observed behavioral and neural difference in the processing of hierarchical structure for the Iranian and Western excerpt. However, the Iranian and Western excerpts presented the same length of dependency between first and final chord, hence similar working memory load. Therefore, the observed significance in recognition of the violation of non-local dependencies cannot merely reflect the working memory process. Considering the obtained results it is more likely that native listeners (without long-term exposure to Western music) can process the complex non-local dependencies in their culturally familiar music more effectively, compared to the Bach's chorale which is not heard in their daily lives. In other worlds, presence of rhythmic structures and melodic fragments that are representatives of Iranian music created a familiar context in which recognition of such complex non-local syntactic structures was feasible for Iranian listeners.

**Table 4:** Behavioral results for the follow up study.

	**Western**	**Iranian**
**GROUP I WITH ORIGINAL BACH'S CHORALE**		
Conclusiveness	−0.06 (0.18)	**−0.42 (0.18)**
Valence	−0.10 (0.19)	**−0.53 (0.16)**
Arousal	−0.08 (0.16)	−0.03 (0.25)
**GROUP II WITH SIMPLIFIED BACH'S CHORALE**		
Conclusiveness	−0.17 (0.21)	**−0.54 (0.23)**
Valence	−0.04 (0.23)	**−0.51 (0.19)**
Arousal	0.02 (0.11)	0.29 (0.20)

*Mean rating values (with standard error in parentheses) of differences between versions (modified subtracted from original). Bold font indicates that differences of ratings between original and modified versions were statistically significant (p < 0.05)*.

Studying the processing of the two musical hierarchical structures, the main electrophysiological findings were for the Iranian excerpt (i) a significant negativity over the central areas during 180–300 ms, which was mostly due to larger negative component in the ERPs of modified excerpts (although not shown here), followed by (ii) a marginally significant negativity during 450–700 ms in the same areas, and (iii) a negativity which manifested globally, although more pronounced in the frontal electrodes, during 750–950 ms which was due to negative component amplitude in response to modified excerpts. No effect of group (musicians vs. non-musicians) was observed. Comparing the processing of the two musical hierarchical structures for the Bach's chorale (original vs. modified versions) the three aforementioned negativities, although observed fell short of significance. The early negativity with its maximum at around 230 ms, which was evoked by hierarchically irregular final chords of the Iranian excerpt, demonstrated an early brain response to the violation of non-local dependency for the culturally familiar music. In the current study the early negativity, although present in the frontal regions bilaterally, reached significance in the central areas. Previous studies reported a bilateral frontal distribution for this early negativity for unattended processing of hierarchical non-local dependencies (Koelsch et al., 2013) and relatively local dependencies (Loui et al., 2005). fMRI Studies have also reported activations in inferior frontal and the superior temporal gyrus for violations of harmonic regularities (Koelsch et al., 2002; Koelsch and Friederici, 2003). The aforementioned early negativity was followed by a later ERP response that marginally reached significance over the same central electrodes. A later ERP response to irregular hierarchical structures manifested globally, although more significant in the anterior regions. The presence of this component was previously reported (Koelsch et al., 2013) with an anterior distribution, and explained through possible processes of harmonic integration. Replication of the ERP findings by Koelsch et al. in the current study suggests that the same mechanism was employed during the processing of nested non-local dependencies of the Iranian excerpt. The observation that the aforementioned ERP responses to hierarchical irregularities did not reach significance for the Bach's chorale indicates that for our subjects processing of non-local dependencies in the context of Western music was not neurally well-established compared to the processing of the same structural dependencies in Iranian music. Since the participants of the study conducted by Koelsch et al. were presumably European listeners familiar with Western music and music from the Baroque era, our findings are indicative of the role of culture in perception and processing of musical syntax.

Oscillatory activity in the beta frequency range (13–25 Hz) was higher for the modified versions of the Iranian excerpts in the time window 150–300 ms. The difference reached significance over the central areas bilaterally. Recently Chang et al. (2016) indicated larger beta band power around 200–300 ms evoked by deviant tones, compared to standard tones. They also noted that this effect was larger when deviant tones were less predicted, suggesting that the effect may be related to prediction processes regarding the timing and content of the upcoming event. Although they reported the aforementioned effect over the auditory cortex through performing source localization the indicated larger beta band power for modified versions in the current study over central areas may be indicative of the same prediction processes involved in comprehension of syntactic structures. One of the limitations of the current study was the absence of recordings from temporal areas, which could provide a better justification of the aforementioned hypothesis.

During the first half of the excerpt global decrease in alpha (8–13 Hz) phase synchrony was evoked by irregular hierarchical structures. An early decrease (in the time window 185–250 ms) in long range alpha phase synchrony has previously been reported during processing of local syntactic irregularities in music (Ruiz et al., 2009) suggestively due to violation of musical expectancy. Further, increase in long-range alpha coupling was observed during music listening (Wu et al., 2012) as well as in mental imagery and mental performance of music (Petsche et al., 1997). Ruiz et al. (2009) provided the first evidence for the integration between the right inferior frontal cortex and left tempro-parietal regions in processing musical syntactic structures. They indicated that deviation of the supertonic chord from the already established harmonic expectations could be the cause for early weaker integration between tempro-parietal brain areas, in charge of early processing stages, with the frontal areas, where the relation between a chord function and the previous context is presumably formed. In the current study alpha decoupling was observed between anterior and posterior regions as well as between hemispheres in the posterior areas for excerpts with hierarchical syntactic irregularities. This phenomenon which occurred in the first half of the excerpt and possibly after early stages of acoustic feature extraction can reflect the mismatch between the lower level processing of the auditory stimuli in the posterior areas (although temporal electrodes were not included in this study) and the long-term memory content in the frontal areas.

The phase synchrony analysis revealed significantly greater theta (4–8 Hz) synchrony for modified than original conditions between left anterior channels and right posterior channels. This coupling along with increased alpha band phase synchrony in the posterior regions was only significant in the second half of the excerpts without any interaction with the factor group. Late increase in long range theta coupling between left frontal and posterior areas was previously observed during semantic priming for unrelated conditions (Mellem et al., 2013). Moreover, fMRI studies have reported recruitment of the left inferior frontal gyrus (LIFG) in addition to posterior cortex as a result of unrelated verbal priming (Badre et al., 2005; Gold et al., 2006). It has been suggested (Mellem et al., 2013), within the context of related fMRI studies, that the observed theta coupling can be an evidence of top down processing; during unrelated priming the LIFG may be engaged to assist the efficient retrieval during this “more difficult situation.” Mellem et al. also proposed another hypothesis for the observed effect suggesting that the observed long range theta coupling may be indicative of increased working memory load during trials of unrelated verbal priming. A more recent study (Meyer et al., 2015) reported late long range theta coherence between left-frontal and left-parietal and between left-frontal and right-inferior-temporal cortices in a verbal working memory paradigm. Their experiment design required retrieval of antecedent nouns from a preceding sentence, hence processing of non-local dependencies in language. The results showed that for the embedded-antecedent condition compared to the non-embedded-antecedent condition coherence between the left-frontal and left-parietal clusters and between the left-frontal and right-temporal clusters was significantly higher. The authors suggested the role of memory retention for the observed effect. It is less likely that during passive listening in the current study the observed late long range theta synchrony was related to working memory processes, since subjects were instructed to pay attention to the concurrently played movie and were not aware of the intention of the experiment. The latency of the increased theta synchrony between left anterior and left posterior areas, accompanied by heightened local alpha phase synchrony as well as oscillatory power in the right posterior region, for the excerpts with hierarchical irregularities may be indicative of the role of higher level processing of these excerpts and increased recruitment of the LIFG for processing of less expected conditions. This view goes in line with a former hypothesis (Mellem et al., 2013) which suggested that top down processing is recruited for processing of unrelated words in language.

Our study only included non-musicians and musicians with training on Iranian music. Recruiting musicians with formal training on Western classical music would provide an opportunity to compare the effect of culturally familiar to culturally unfamiliar music for this group of musicians. Moreover, we only used one Western and one Iranian excerpt. A study with different culturally familiar and culturally unfamiliar music excerpts is required to further investigate the role of culture in processing of musical syntax.

## Conclusions

We presented Western and Iranian musical excerpts with hierarchical structures to investigate the role of culture in perception and processing of complex musical structures. We replicated previous findings (Koelsch et al., 2013) for neural manifestation (in terms of ERP responses) of violations in non-local dependencies for our Iranian excerpt. The effect was however absent for the Western music. We propose that for societies repeated exposure to cultural music creates a feeling of knowing and ease toward one's own musical system which facilitates perception of complex musical structures, e.g., non-local embedded dependencies, in the context of culturally familiar music compared to culturally unfamiliar music.

## Author contributions

SM and HA designed the experiments. HA conducted the experiments, and analyze data. SM supervised data analysis. HA and SM prepared the manuscript.

### Conflict of interest statement

The authors declare that the research was conducted in the absence of any commercial or financial relationships that could be construed as a potential conflict of interest.
