# Supplementary material for: Culture Modulates the Brain Response to Harmonic Violations: An EEG Study on Hierarchical Syntactic Structure in Music
Source: Front Hum Neurosci. 2017 Dec 6;11:591. doi: 10.3389/fnhum.2017.00591 (PMC5723651; doi:10.3389/fnhum.2017.00591)
Supplement: Supplementary file 1 [file Presentation1.PDF]

Supplementary Material

Culture modulates the brain response to harmonic violations: an EEG study on hierarchical syntactic structure in music

Haleh Akrami, Sahar Moghimi*

*** Correspondence:** Sahar Moghimi: s.moghimi@um.ac.ir

1 Supplementary Audios

Audio File A1. Original version of the Iranian excerpt used in our experiment in key G major**,** score is provided in **Figure 1 (A)** (Main text).

Audio File A2. Original version of J. S. Bach's chorale "Liebster Jesu, wir sind hier in key G major, score is provided in **Supplementary Figure s.2 (A).**

Audio File A3. Simplified version of J. S. Bach's chorale "Liebster Jesu, wir sind hier in key G major, score is provided in **Supplementary Figure s.2 (B).**

Audio File A4. Modified version of **Audio File A1** in key G major.

Audio File A5. Modified version of **Audio File A2** in key G major.

Audio File A6. Modified version of **Audio File A3** in key G major.

2 Supplementary Figures and Tables

2.1 Supplementary Figures

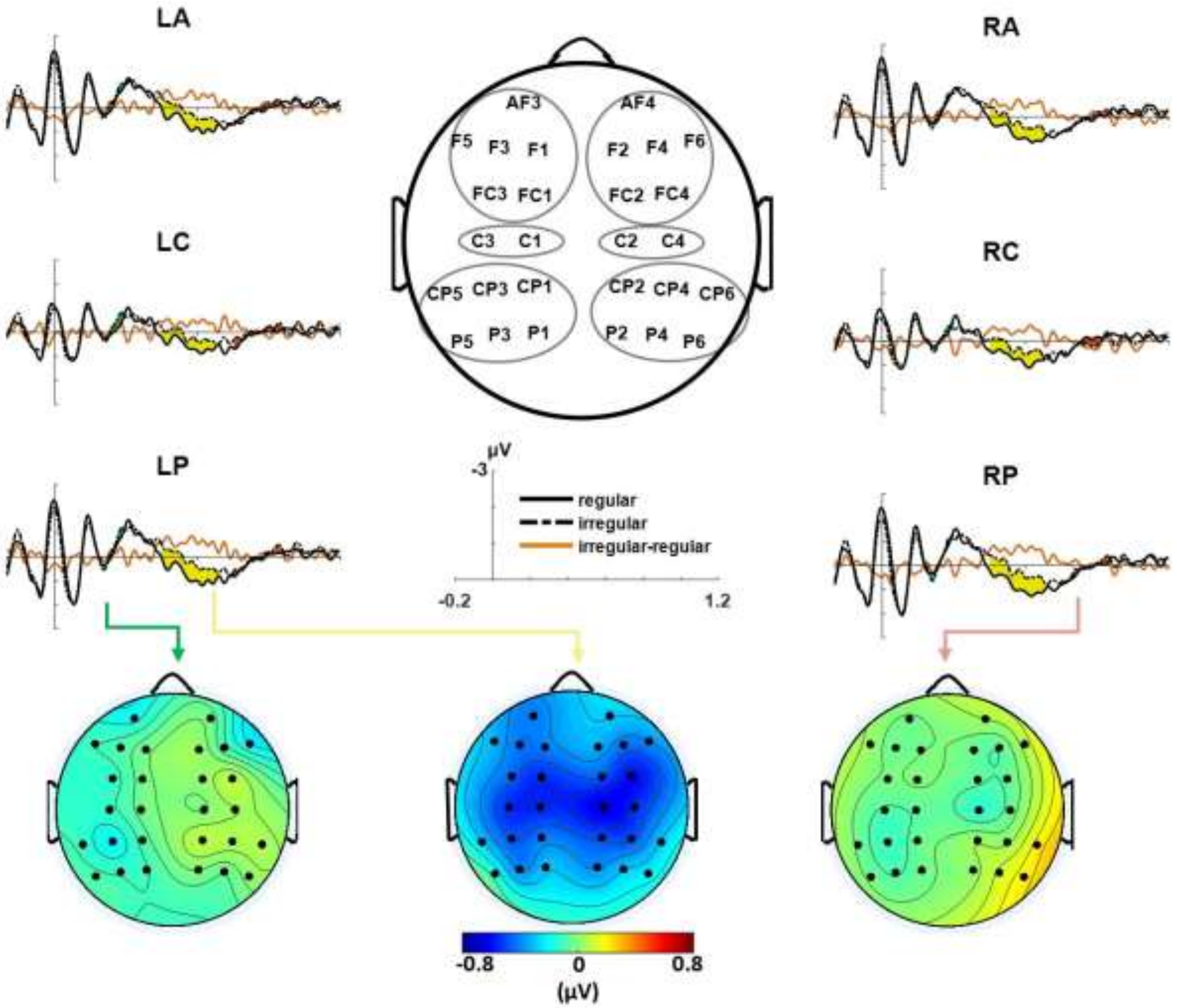

Supplementary Figure s.1. Event-related brain potentials (ERPs) evoked by the final chords in the ROIs are shown (original versions of Western excerpt subtracted from the modified versions). Bottom figure shows the scalp distribution of the time windows that evoked significant effects in the Iranian piece.

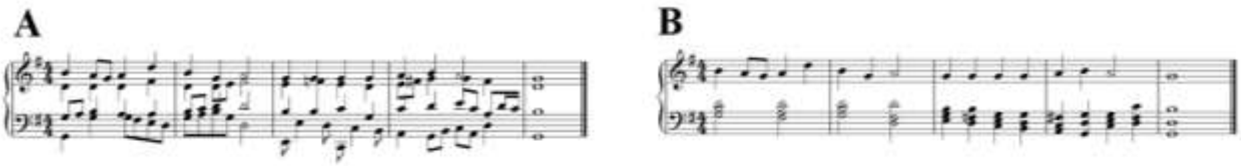

Supplementary Figure s.2. (A) Original version of the J.S. Bach's chorale BWV 373 (B) The simplified version of J.S. Bach's chorale BWV 373

2.2 Supplementary Tables

Supplementary Table s.1- Mean ERP differences between two versions (original subtracted from modified) for the Iranian excerpts (with SE in parentheses) are shown for the central region (averaged over the two central ROIs). Bold fonts indicate that differences of ERP waveforms were statistically significant ($p<0.05$). * $p<0.01$

Time Window	Mean Difference (SE)
0-100	0.13(0.26)
100-200	-0.03(0.22)
**180-300**	**-0.40(0.19)**
**200-300**	**-0.45(0.21)**
300-400	-0.04(0.21)
400-500	-0.16(0.32)
500-600	-0.52(0.31)
600-700	-0.45(0.27)
700-800	-0.27(0.24)
**800-900**	**-0.83*(0.23)**
**750-950**	**-0.64*(0.22)**
900-1000	-0.35(0.26)
1000-1100	-0.20(0.24)
1100-1200	-0.39(0.30)

Supplementary Table s.2- Mean ERPs differences between two versions (original subtracted from modified) for the Western excerpts (with SE in parentheses) are shown for the central region. No statistically significant difference was observed in any of the time windows.

**Time Window**	
0-100	-0.13(0.19)
100-200	-0.03(0.31)
200-300	-0.45(0.35)
300-400	-0.29(0.35)
400-500	-0.51(0.32)
500-600	-0.50(0.30)
600-700	-0.48(0.30)
700-800	-0.45(0.27)
800-900	-0.34(0.22)
900-1000	-0.40(0.29)
1000-1100	-0.17(0.33)
1100-1200	-0.11(0.22)
